# A Therapeutic Need

**Published:** 1899-08

**Authors:** 


### A THERAPEUTIC NEED.

IT REQUIRES no prophet to see indications of the greater prominence that therapeutics is to attain in the next decade. The impetus given by research and discovery along the line of specific treatment of disease, has been decided. But while the medical world is first dazzled then stimulated by such a discovery as that of the anti-toxins, we are convinced that a large part of the foundation of a sounder therapeutics must be a more intimate knowledge of our well-known older drugs and of their influence upon the diseased body.

It appears to us, therefore, to be especially needful that of our book of standards, the U. S. Pharmacopeia, shall possess the necessary resources for the progressive therapeutist in respect to old drugs as well as new. Interest in the coming revision of this book is increasing. There is properly much thought as to what shall be admitted; but there should be more regard for the character, as to uniform activity, of the preparations of drugs, which for years have been known to be valuable.

The lines of construction of a pharmacopeia of national authority must necessarily be conservative. Nothing of uncertain character can be admitted, or its value as a standard is destroyed. For the same reason its scope is a restricted one. Greater, there fore, the need of bringing it at each revision up to the mark of most advanced scientific progress, in view of the long interval of ten years between additions. In addition to the incorporation of details which have been matured, each edition should mark the recognition of new principles that can "serve as guides" to progress in pharmacology until the succeeding revision. Thus in the last edition (1890) the principle of standardisation of preparations by chemical assay was recognised in its application to cinchona, nux vomica and opium. To demand that this principle be now applied to other drugs just as far as is possible, with due regard to practicable methods, is the right of the profession. Particularly so, because the uncertainty of drug activity has always presented a hindrance to scientific therapeutics. Efficient medicinal treatment requires not only a knowledge of drugs and their application, but first of all uniformly active drugs. In respect of furnishing such, no doubt the large manufacturer, who can assay by elaborate methods, has an advantage over the retailer with moderate equipment; and so far as his methods are ethical and scientific they may be competent to lead the pharmacopeia in lines of progress. Certain it is that the authors of the next revision will have to face the question of recognising another principle—namely, that of standardisation by other than chemical tests. No one will question the propriety of recognising antidiphtheritic serum, but it cannot be standardised by chemical means. The same is true for the present of digitalis, ergot and cannabis indica particularly, whose activities are greater than those of any of their isolated constituents.

One manufacturing firm has given an impetus to the method of physiological testing of drugs and has done commendable work in that direction; and when it is reported that large quantities of drugs have been found inactive, we may well question the value of many of the galenical preparations dispensed.

The need of more reliable drug preparations is imperative, and if we cannot look to our pharmacopeia to fix standards of activity its usefulness will be on the decline.

---

THERE has been so much notoriety attached to christian science and its "healers" the past month or two that an exposé of the teachings of the seers of the cult will be particularly interesting at this time.

In almost every daily newspaper one picks up nowadays one sees reports of deaths under the beneficent ministrations of the so-called "healers," or maybe a death-bed denunciation of the healers and their methods, or a report of a sorrow-ridden family's anguish over the death of a child of tender years, whom whooping cough had claimed while a healer sat by and looked wise.

In Buffalo, too, there are now evidences of the christian science evil. On another page reference is made to Dr. Wende's attitude, and to the women who have rushed into print. The Saunders case is too recent to need more than a passing mention.

The exposé printed elsewhere is an illustration of the good a daily newspaper can do when it gets on the right track. The New York *World* sent one of its reporters out to learn the truth and published it. How well he succeeded may be learned by reading the first article published in this issue of the JOURNAL, which is a careful resumé of the *World's* exposé.

---

BY MEANS of a cable dispatch to the newspapers from London, it is learned that Dr. Lambert Lack, the eminent throat specialist, who is honorary surgeon to the Golden Square Throat Hospital, has discovered the cause of cancer, the will-o'-the-wisp of the medical fraternity.

Dr. Lack, the cable says, has been studying the question for years, and has learned that the "disease invariably began with an injury of a particular character to what is known as the basement membrane of the mucous membrane and its allied structures.

"Having arrived at this important truth, he set to work to produce cancer in the lower animals, and succeeded."

The whole subject matter was presented to the London Pathological Society by Dr. Lack, and by that society was thoroughly investigated. The result of the deliberations was an unqualified approval of the discovery.

Dr. Lack the cable also says is now experimenting with a serum for the cure of cancer under the auspices of the London society.

This is the second cancer discovery reported within a few months. A while ago Dr. Bra, of Paris, claimed to have isolated the cancer germ, but his discovery was discredited by Professor Curtiss of Lille.

Mayhap the Buffalo laboratory will have a discovery to announce shortly if the state authorities are patient and will continue the appropriation for the maintenance of the work.

---

The Buffalo *Evening News* has taken at last a common sense view of christian science controversy and makes this suggestion in a recent issue :

WHY NOT TAP THE FEES.

All the objections of the christian science people to the proposed ordinance seem to be based on the alleged interference with "deeds of mercy." They can't think of stopping people from caring for sick neighbors. Nevertheless, scientist healers tax their clients for the biggest kind of fees.

Why not amend the ordinance so as to cut off fees. Let neighbors attend neighbors all they like, but when one takes fees for treatment of disease and has no legally established qualifications for so doing make the punishment heavy and enforce it with rigor.

That will separate the money-makers from the doers of merciful deeds in short order.

This will have an excellent effect on the fakirs. It is well known they do not heal for sweet charity's sake. All they do is for the glory of God and the almighty dollar. It is part of their teaching not to heal without pay.

---

At last the newspapers are becoming aroused to the evils that result from the practice of medicine by so-called christian scientists. The Journal has no quarrel with these misguided people so long as they restrict their ministrations to the religious side of their work; but when they assume to cure bodily ailments it is quite another question.

The New York *Tribune* recently published an editorial on this subject, which abounds with such good sense that we take pleasure in giving it space, as follows:

THE PRACTICE OF MEDICINE.

Let us leave in abeyance for a moment the general question of the efficacy of so-called "christian science." Let us recognise that opinions differ as to its scientific character, and as to its Christian character, and as to whether it ever does any good. The present point of consideration is its legal standing under the laws for the regulation of the practice of medicine. Christian scientists have all along contended that they are not amenable to the law, because, as they do not administer drugs and do not charge fees, they do not practise medicine. We observe that a coroner in Philadelphia sustains that view of the case, and charged his jury the other day to that effect.

That contention is a paltry quibble. So far as the charging of fees is concerned, it is perfectly well-known that christian scientists do in some cases charge regular fees, and in many cases, whether they charge them or not, receive them. Moreover, many regular doctors of medicine treat many patients free of charge. Are they not practising medicine when they do so? Is not the practice of medicine conducted at dispensaries and in the free wards of hospitals? Are we solemnly to assert that to prescribe ipecac and charge a dollar is to practise medicine, while to prescribe ipecac and charge nothing is not to practise medicine?

So far as the other point is concerned, relating to the administration of drugs, the quibble is equally obvious. The practice of medicine does not mean the giving of drugs. It means the healing of ailments by whatever means. The doctor who tells the patient to take more exercise, or to eat less meat, or to wear flannel, practises medicine in so doing just as much as though he prescribed quinine or blue pills. Every man who knows the meaning of English words knows that to be the case. What, then, is the use of saying that christian science is not the practice of medicine simply because it administers no drugs?

To do so is to plead the baby act, or to assume that all men are fools. It is time to stop such nonsense. Christian science ought to stand on its own merits, and be responsible for its doings, in an honest, straightforward fashion, and not try to shield itself behind a disingenuous quibble.

---

THE Buffalo Evening *News* recently made appropriate comment upon this misguided cult and its ridiculous opposition to the municipal ordinances in the following well-chosen words:

### DANGEROUS PEOPLE.

Last night's hearing of the christian science ordinance made one thing clear.

The scientists refuse to be bound by the laws that govern everybody else in the community. They will not report cases of contagious disease, as every physician is required to do, and as in fact every person in the community is required to do, whether treating the disease or not.

The scientists thus abandon the claim of persecution which has been their stand by. They are not willing to abide by the laws that govern others. Their statement that this ordinance is being framed to catch them is true. They require special legislation because they defy the existing law, and by so doing spread disease.

Cases were cited last night where the scientists had sent children to school with contagious diseases, denying that there was contagion. There can be no safety for any one in the community when such practises are allowed. The scientists are dangerous people. They should be restrained by law from imperiling the health of the public by concealing cases of contagious disease.

---

THE report of the State Board of Medical Examiners of the examinations held in May, 1899, exhibits statistics as follows :

| | | |
|---|---|---|
| Total number of candidates | 131 | |
| Successful | 111 | 84.73 per cent. |
| Unsuccessful | 20 | 15.27 " |

| | | | |
|---|---|---|---|
| Rejected in anatomy | 12 | Rejected in one topic | 10—10 |
| " " physiology and hygiene, | 3 | " " two topics | 3— 6 |
| " " chemistry | 7 | " " three " | 1— 3 |
| " " surgery | 3 | " " four " | 3—12 |
| " " pathology and diagnosis, | 9 | " " five " | 3—15 |
| " " therapeutics, practice, etc | 9 | " " six " | 0— 0 |
| " " obstetrics | 3 | " " seven " | 0— 0 |
| | 46 | | 20—46 |

| | |
|---|---|
| Highest general average | 95.3 per cent. |
| Total number "honor" men | 10 |

